# High-Resolution Chrono-Transcriptome of Lactococcus lactis Reveals That It Expresses Proteins with Adapted Size and pI upon Acidification and Nutrient Starvation

**DOI:** 10.1128/aem.02476-21

**Published:** 2022-04-13

**Authors:** João P. C. Pinto, Rutger Brouwer, Araz Zeyniyev, Oscar P. Kuipers, Jan Kok

**Affiliations:** a Department of Molecular Genetics, Groningen Biomolecular Sciences and Biotechnology Institute, University of Groningen, Groningen, the Netherlands; University of Tartu

**Keywords:** DNA microarrays, *Lactococcus lactis*, acidification, nutrient starvation, physiology, transcriptomics

## Abstract

Whole-genome transcriptional analyses performed on microorganisms are traditionally based on a small number of samples. To map transient expression variations, and thoroughly characterize gene expression throughout the growth curve of the widely used model organism Lactococcus lactis MG1363, gene expression data were collected with unprecedented time resolution. The resulting gene expression patterns were globally analyzed in several different ways to demonstrate the richness of the data and the ease with which novel phenomena can be discovered. When the culture moves from one growth phase to another, gene expression patterns change to such an extent that we suggest that those patterns can be used to unequivocally distinguish growth phases from each other. Also, within the classically defined growth phases, subgrowth phases were distinguishable with a distinct expression signature. Apart from the global expression pattern shifts seen throughout the growth curve, several cases of short-lived transient gene expression patterns were clearly observed. These could help explain the gene expression variations frequently observed in biological replicates. A method was devised to estimate a measure of unnormalized/absolute gene expression levels and used to determine how global transcription patterns are influenced by nutrient starvation or acidification of the medium. Notably, we inferred that L. lactis MG1363 produces proteins with on average lower pIs and lower molecular weights as the medium acidifies and nutrients get scarcer.

**IMPORTANCE** This data set is a rich resource for microbiologists interested in common mechanisms of gene expression, regulation and in particular the physiology of L. lactis. Thus, similar to the common use of genome sequence data by the scientific community, the data set constitutes an extensive data repository for mining and an opportunity for bioinformaticians to develop novel tools for in-depth analysis.

## INTRODUCTION

With the availability of increasingly more genome sequences of the industrially important and well-characterized bacterium Lactococcus lactis (1–10), postgenomics experimental approaches such as transcriptomics, proteomics, and metabolomics analyses have robustly been applied to this organism. Also, from the resulting data sets and aided by specifically designed bioinformatics and modeling tools, systems biology holistic approaches have greatly improved our understanding of this lactic acid bacterium (LAB). Together, these methods have allowed improvement of genome annotation and yielded valuable information for the construction of complete metabolic network models (see, for examples, references 11 and 12). However, the dynamics in gene expression necessary for keeping homeostasis during growth and adaptation to natural stresses caused by intrinsic and extrinsic factors such as changing nutrient availability, oxygen pressure, or pH have not yet been studied in great depth.

Whole-genome transcriptional analyses are traditionally based on a small number of samples (see, for example, reference 13). However, some transitions in gene expression could in principle be fast and transient, as indeed is shown in this report, resulting in quick bursts of expression or fast oscillations throughout the growth curve. Moreover, transition points between the classical growth phases could be sharp and might be missed when sampling is not frequent enough. As also demonstrated for Bacillus subtilis (14), a detailed profile of transcriptional fluctuations in expression of each gene during growth of a bacterium that is normally grown in batch fermentation is desired in order to obtain a rich repository of the expression profile of each gene in the genome. Such information allows extensive and in-depth mining for correlations or anticorrelations between genes regarding their temporal expression. Moreover, it may help to clearly define growth phases in terms of signatures of gene expression and possibly identify subphases within and between the growth phases.

In this study, we have developed an experimental design that charts the often rapid transcriptional changes of genes of L. lactis. A batch-grown culture of the widely used model organism L. lactis MG1363, grown in GM17 broth at 30°C, was sampled every 15 min. The transcriptomes of the 45 samples obtained were analyzed using the relatively cost-effective DNA microarray technology. The results show, among others, that gene expression patterns can be used to unequivocally distinguish the different growth phases in L. lactis batch fermentation. In addition, several short-lived transient gene expression patterns were identified that could, e.g., help explain gene expression variations frequently observed in biological replicates in microarray experiments. The entire data set, which we believe to be a rich resource for the microbial research community, is available in the supplemental material.

## RESULTS AND DISCUSSION

### Growth of Lactococcus lactis MG1363 in GM17.

**FIG 1:**
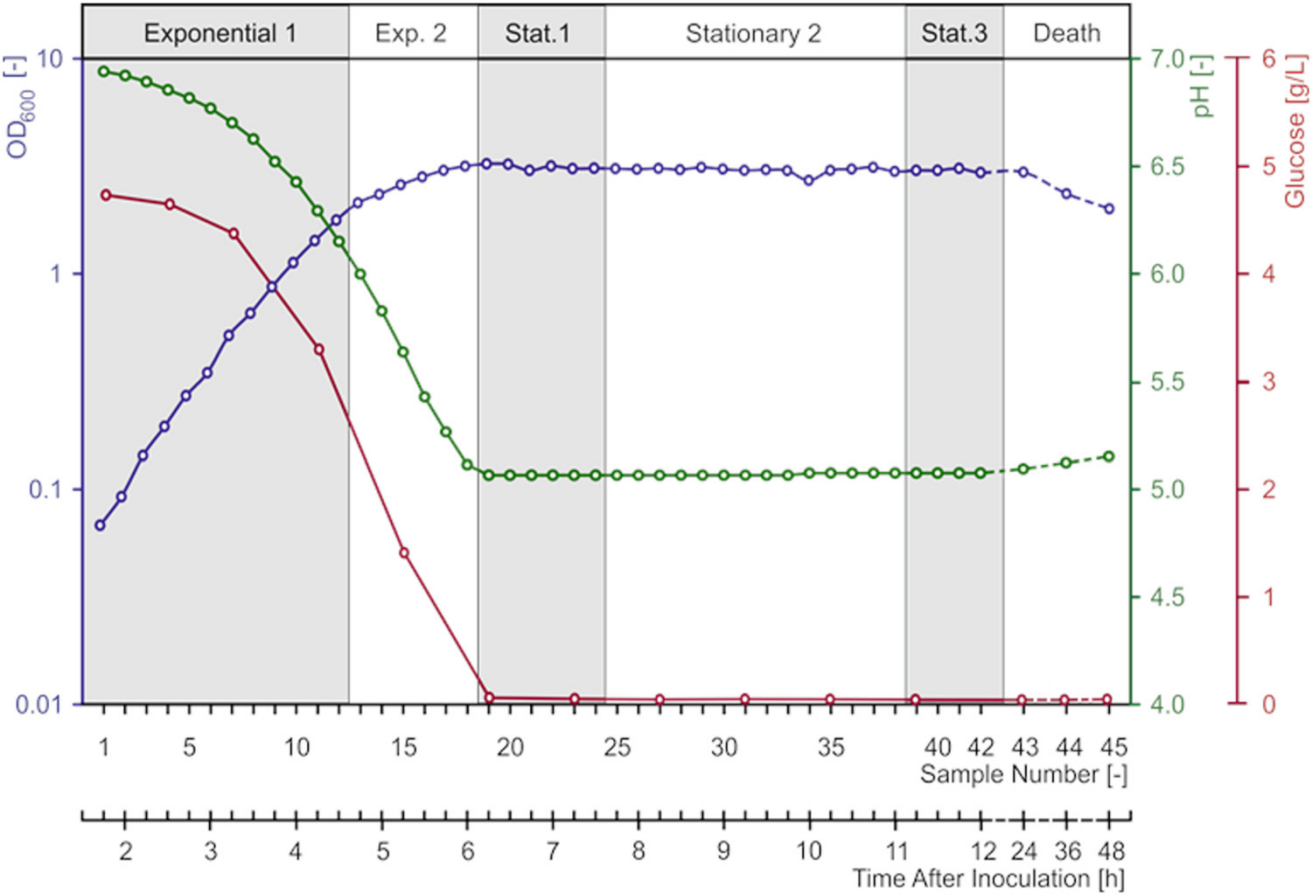
Growth of L. lactis MG1363 in GM17. OD_600_, pH, and dissolved glucose in the medium were recorded throughout the batch fermentation at 30°C. Gray and white bands indicate the different growth phases as indicated on the top axis. Exp., exponential; Stat., stationary.

A 12-L GM17 batch fermentation was initiated with a 1% inoculum of an exponentially growing culture of L. lactis MG1363 growing in the same medium (see Materials and Methods). The absence of an adaptation (lag) phase led to a well-defined and reproducible growth curve, with minimal interference of the history of the inoculum on the growth of cells during the fermentation (Fig. 1). The reproducibility of growth using this procedure was confirmed by repeating the fermentation procedure 3 times (data not shown). Growth arrest occurred at an optical density at 600 nm (OD_600_) of 2.8 because of depletion of glucose (0.5% [wt/vol] at the start of the experiment) from the medium (Fig. 1). This metabolic constriction and its consequent arrest of the production of lactate also coincided with the stabilization of the pH of the medium at a value of 5 (Fig. 1). It should be noted that for this lactic acid bacterium, glucose is limiting when its initial concentration is below approximately 0.75%. When higher concentrations of glucose are used, cells keep growing to a higher OD, with consequent higher acidification of the medium. In those circumstances it is the further decrease in pH that limits growth, as a result of lactate not being neutralized (data not shown). At around 1.5 h after inoculation, the culture became sufficiently dense to allow sampling of cell material for the DNA microarray procedure. Samples were taken every 15 min during the first 12 h, after which three samples were taken at 24, 36, and 48 h after inoculation. Subsequently, the samples from 1 fermentation were examined by DNA microarray analysis (see below).

Exponential growth was characterized by two discernible phases, each with a distinct growth rate (Fig. 1). Optical density remained constant between 6 and 24 h after inoculation. However, whole-transcriptome analyses suggested the existence of three distinct periods in the stationary phase (see below). After 24 h, the optical density began to decrease, corresponding to the death phase in bacterial growth. At the same time, medium pH increased marginally, suggesting that since intracellular pH is likely to be higher than that of the medium even during this stage (15), the observed lysis of part of the culture slightly neutralized the medium (see, for example, reference 16 for more on autolysis of L. lactis MG1363).

### Dimensioning-noise-amplitude (D-N-A) scaling.

**FIG 2:**
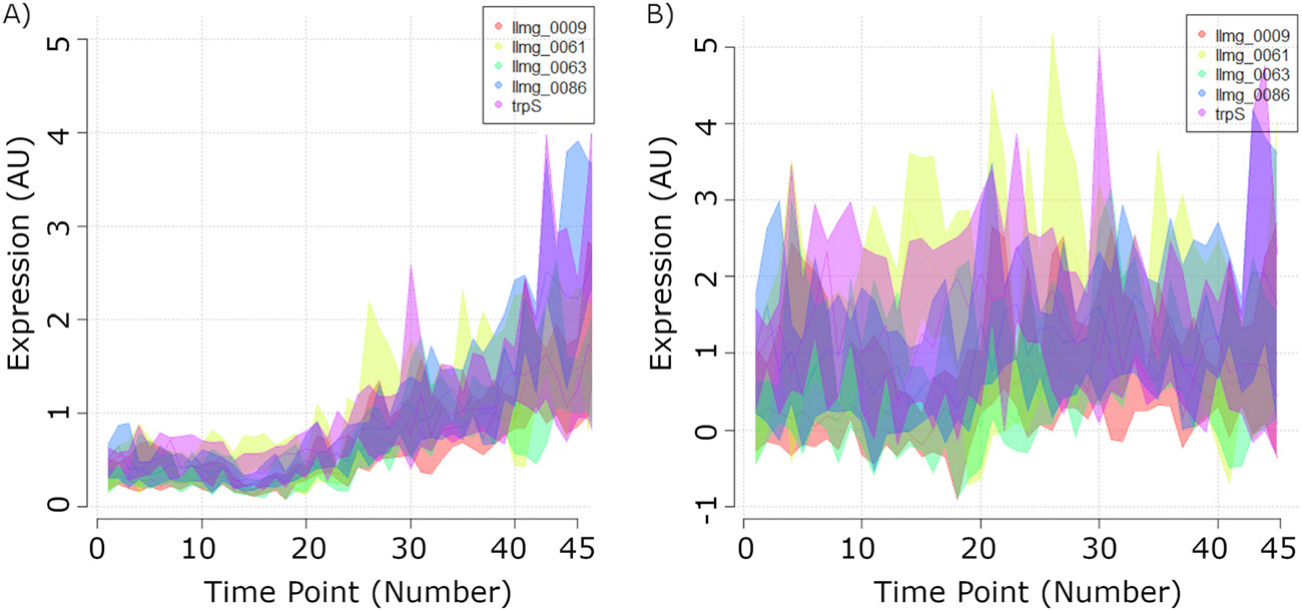
Comparison between standard LOWESS and dimensioning-noise-amplitude scaling. (A) A sample of 5 out of 368 genes that, despite being the least expressed throughout growth, displayed a significant signal increase because of the standard LOWESS interslide scaling. (B) The signal from the same 5 genes after D-N-A scaling. The gene expression plots illustrate the range of expression signals obtained for each gene at each time point, while the darker line within each range represents the median value. AU, artbitrary units.

Standard normalization and scaling of gene expression microarray data typically assume the preservation of the expression levels of most genes, an assumption obviously not true for the entirety of the growth curve. This approach improperly transformed values in a way that made the average of all signals remain roughly constant at all time points (see Materials and Methods). This artificially pushed the values of the last time points (in the death phase) toward a plateau not consistent with, e.g., cDNA synthesis yields (typically 10 times lower in the death phase than the average cDNA yield of all other samples [data not shown]). As a result, when using standard normalization and scaling methods, even genes that were least expressed throughout the growth, with microarray signal intensities in the order of magnitude of what should be considered noise, showed high expression levels toward the end of the experiment (Fig. 2).

To correct for this artifact, we aimed to identify an internal standard that could be used to set the correct level of gene expression data for each time point. It was observed that a significant number of genes, which we hypothesized were not being expressed at a certain time point, did display extremely minute signals with a distribution that typically fluctuated within a specific interval. The signal ranges of the least expressed genes, at each time point, were equivalated to a measure of technical noise and set it to have a constant dimension throughout all data points (arbitrarily equal to 1). Subsequently, these signal ranges were used to recalibrate the entire set of data of all genes, independently for each time point. To implement this analytical process, the genes of which the expression was always below the median in all the time points were initially screened. The signals of a set of 368 genes (a representative selection of which is given in Fig. 2) were set aside as an initial measure of what was considered “noise” range. Due to the stochastic nature of noise, the entire set of 368 gene expression profiles was not used, but rather the 30th and 90th percentile signals from each time point of genes within this group. Less strict cutoffs would lead to the integration of abnormal readings, while stricter cutoffs would also produce a less robust range as a result of eliminating a greater portion of those expression profiles. Also, for each time point, these values were averaged with the ones from the two adjacent time points, to produce a robust proxy of the dimension of noise. This information was then used to transform signals of all genes in the following manner: the mentioned 3-time-points moving average of the 30th percentile was subtracted from all signals for each time point and then the resulting value was divided by the 3-time-points moving average of the 90th percentile. The outcome of this transformation on the so-called background gene expression signals is illustrated in Fig. 2. In this new scale, gene expression is not entirely arbitrary but a relative measure against noise (an expression value of 2 represents roughly twice the average value of noise).

**FIG 3:**
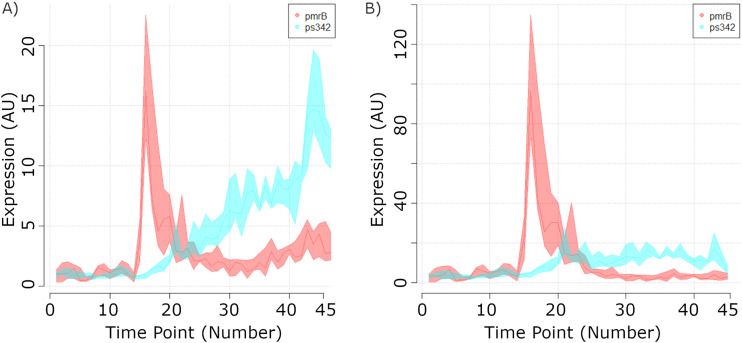
An illustration of the transformation performed through dimensioning-noise-amplitude scaling. (A) Expression profile of *pmrB* and *ps342* after standard interslide scaling. (B) Expression profile of *pmrB* and *ps342* as computed after D-N-A scaling.

This method does not merely lead to a minor correction of the data; it also has a fundamental impact on the interpretation of those data. For example, one could be led to believe that the prophage gene *ps342* is highly expressed toward the end of the stationary phase, at a level that compares, e.g., to the peak in *pmrB* expression just before cells stop growing exponentially (Fig. 3). According to data obtained after D-N-A scaling, although *ps342* indeed starts to be transcribed during stationary phase, the magnitude is certainly not comparable to the peak expression of *pmrB* (Fig. 3).

### Whole-transcriptome analyses.

**FIG 4:**
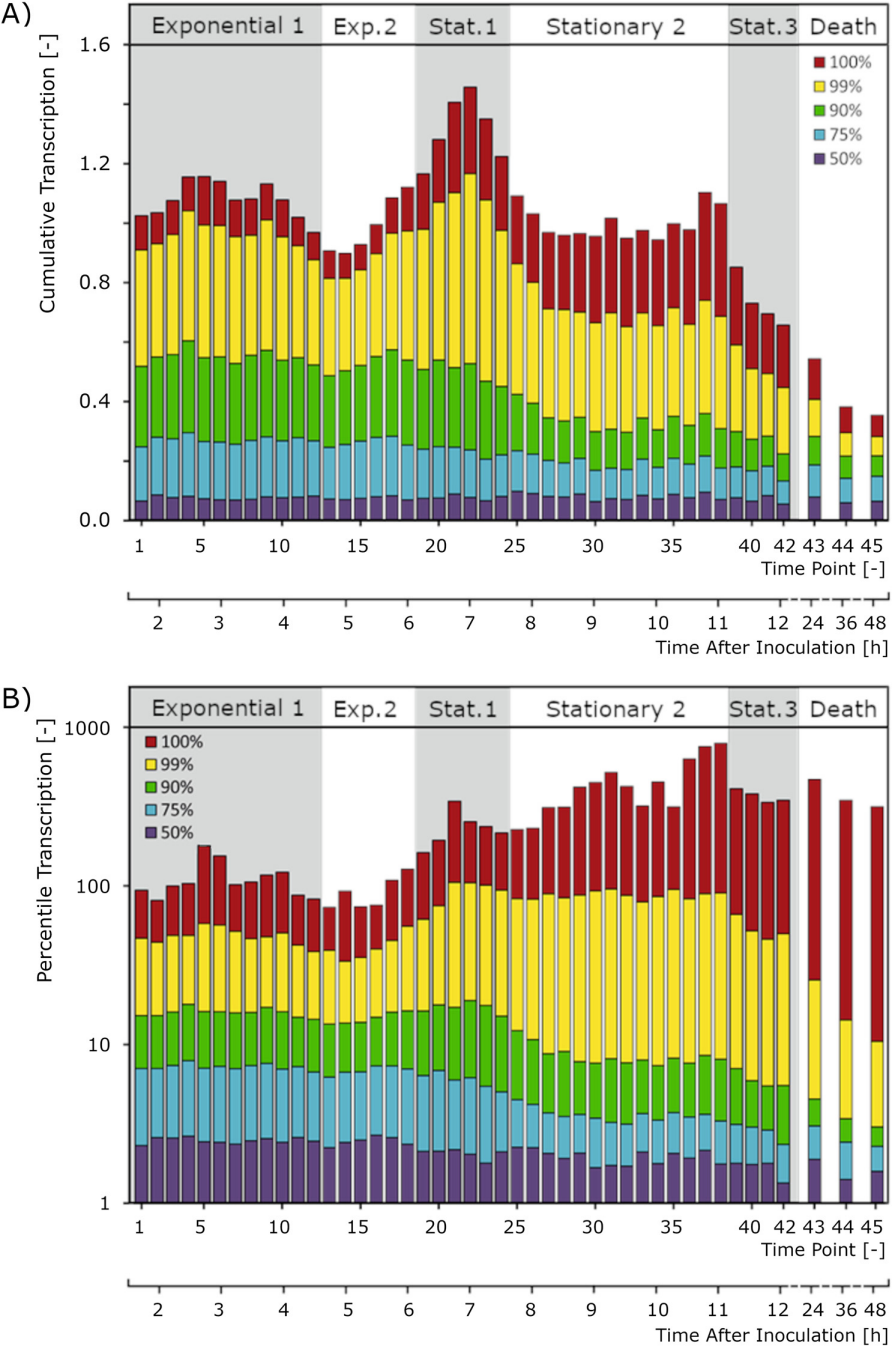
Inferred global transcription (A) and expression percentile (B) levels throughout the growth of L. lactis MG1363 in GM17. For each time point, expression levels were ordered, and percentile cutoffs were used as follows. (A) Cumulative transcription sums the transcription levels of all genes below the percentile cutoff (note that the 100% value offers a measure of the total expression level in the cell at each given time point). (B) Percent transcription measures the transcription level of the gene at that percentile (note that the 100th percentile value offers a measure of the level of expression of the most expressed gene at that time point). Gray and white bands: growth phases as indicated on the top axis and presented in Fig. 1.

As described above, a novel method was developed to capture a sense of the absolute levels of expression of genes throughout growth. Obviously, a difference exists between the level of transcription and measured cDNA signal (for example, the lifetimes of different mRNA molecules vary, and so do their cDNA synthesis yields), but such distinction is generally not made in this text, to be concise. It is clear that the global transcription activity remains roughly constant until the moment that a swift decrease is seen in the growth rate, around time points (tp) 12 and 13, around 4.5 h after inoculation (Fig. 1 and 4). In a second stage, here discriminated as exponential phase 2 since the optical density of the culture is still increasing logarithmically (albeit at a slightly lower rate), the overall transcription level initially decreases but quickly rises as the culture approaches the stationary phase, around tp 18 and 19, a little over 6 h after inoculation.

Glucose depletion and the consequent growth arrest were accompanied in a first stage by a peak in expression around 40% greater than what was observed globally during exponential growth (Fig. 4). This reveals that cells undergo a substantial adaptation process in the initial period of growth arrest. Although the stationary phase, in the standard sense that optical density does not change, lasted until 24 h after inoculation, three distinct stationary subphases were discernible based on the overall transcription level of the cells (Fig. 4). In the initial stationary phase, termed 1 (tp 19 to 24), global transcription peaks but later decreases to a plateau of transcription similar to that observed during the exponential growth period, in stationary phase 2 (tp 25 to 38). Some 5 h after glucose depletion (from tp 38 onward), global transcription levels suddenly drop to what likely represents an energy-saving mode a well as the onset of a phase in which cells fail to maintain essential processes that ultimately preclude viability. As global transcription levels decrease, culture optical density is initially maintained (stationary phase 3; tp 39 to 42), but ultimately, the cells start lysing. This final period, 24 h after inoculation and onwards, constitutes the death phase (tp 43 to 45). As expected, the total culture in this phase shows a reduced transcriptional activity that is 4 times lower than the culture’s peak activity in transcription.

At any given moment in growth, the variable set of the top 10% of most expressed genes accounts, on average, for 50% of the transcriptome of L. lactis (Fig. 4). The top 20% of most expressed genes at any given moment corresponds to roughly 80% of all transcription, which is yet another example of the seemingly universal Pareto principle (also called the 80-20 rule).

Of the 2,308 genes represented on the DNA microarray slides, only 368 were expressed below the median expression per time point throughout growth. As the expression of some of these genes might not have been measured accurately due to inefficient DNA microarray probes, this estimate may be overstated. This set represents less than 20% of all L. lactis genes. Thus, most genetic networks are active at least at some stage during growth of L. lactis in batch culture in a complex medium, where cells go through distinct stages of growth because of changing conditions, such as nutrient starvation and medium acidification.

**FIG 5:**
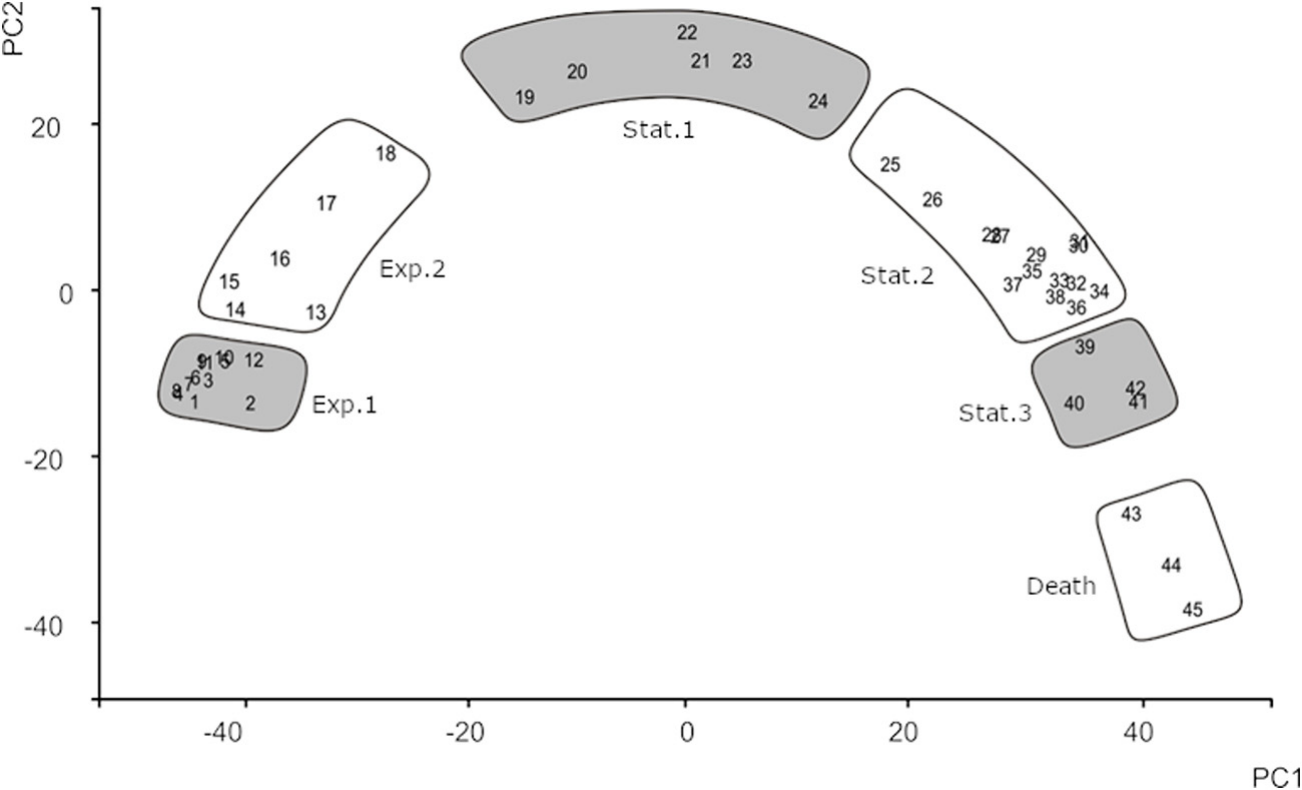
Principal-component analysis. PCA was applied to all gene expression data. All time points were mapped in relation to the two most relevant principal components (PC1 and PC2). Gray and white boxes indicate the different growth phases.

Principal-component analysis (PCA) was used to qualitatively assess the variation in the DNA microarray samples. Remarkably, PCA supports the partitioning of growth phases, as described above, according to growth rates and global levels of transcription. The first two principal components (PCs) account for approximately 60% of all variation in the data set and enable separation of the samples according to the growth phase from which they were taken (Fig. 5). Samples 1 to 12 (exponential growth phase 1) group close together using the first two PCs. The great resemblance of the transcriptomes throughout these initial time points is probably the result of using an inoculum that was kept growing exponentially for approximately 40 h and the consequent absence of a detectable lag/adaptation phase. After exponential phase 1, the profile of gene expression drifts continuously as a result of the adaptations that the cells undergo. Later, a few time points into stationary phase 2, cells again seem to stabilize their transcription profiles, since tp 27 to 38 cluster close together (Fig. 5). Subsequently, and while the culture optical density is still constant, the collective transcription pattern is further modified (tp 39 to 42). The last 3 time points are characterized both by a decrease in the OD_600_ and a drift regarding the gene expression profile.

### The time series as a reference curve.

**FIG 6:**
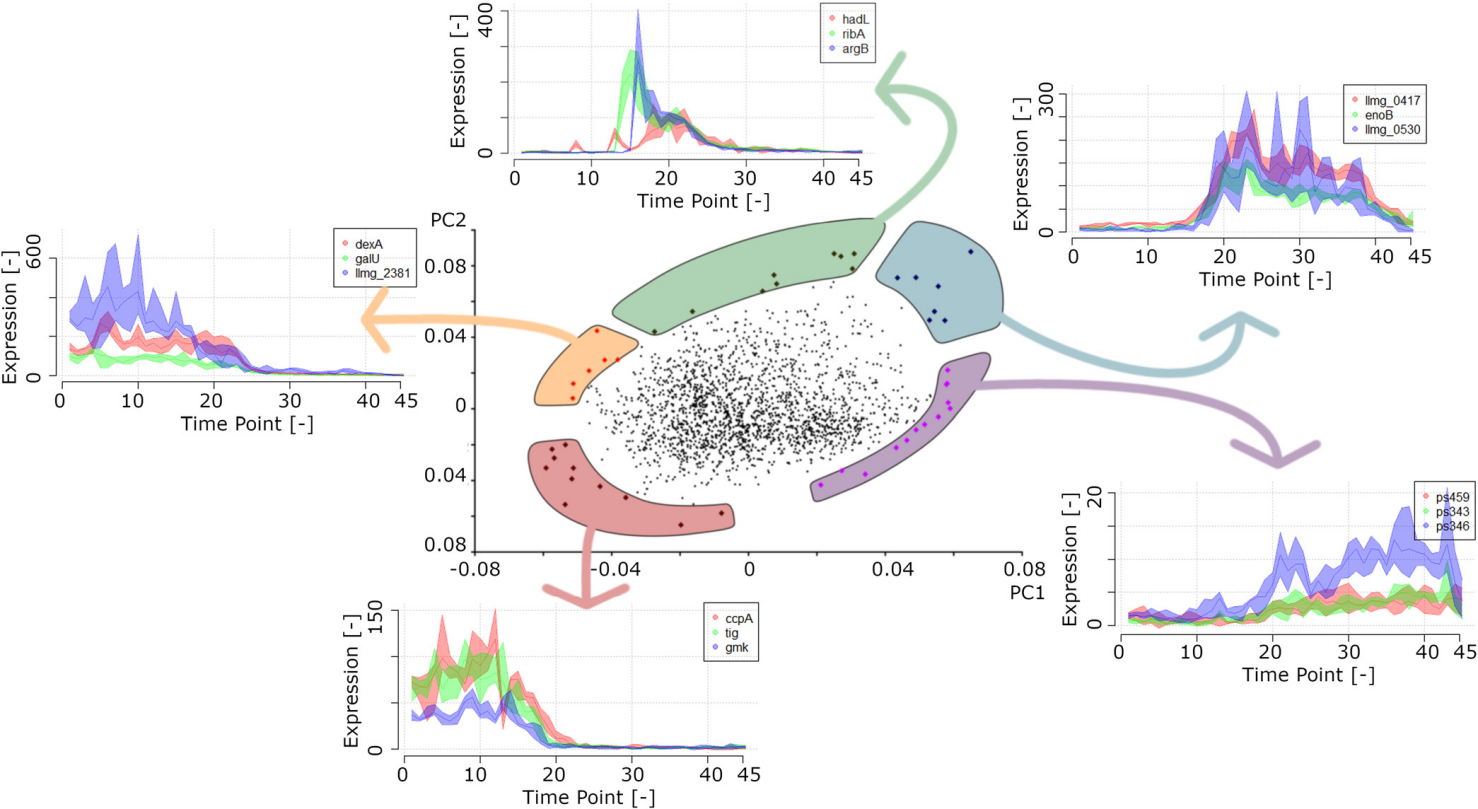
Identification of growth phase markers. Genes that contributed the most to PC1 and PC2 constitute optimal markers for growth phase discrimination since they are expressed only during specific periods of growth.

An immediate application of the transcriptome data reported here follows from the PCA described above. Genes of which the expression contributed strongly to separate the various time points according to their expression profiles are characterized by well-defined periods of expression (Fig. 6). This information allows, for example, the design of growth phase markers or validating and interpreting other DNA microarray data using this time series (chrono)-transcriptome data set as a reference.

Using the expression profiles of the 45 growth phase marker genes selected from the PC analysis, one can validate the growth stage of an L. lactis culture. Most often, samples from bacterial cultures are taken during the mid-exponential or mid-stationary phase of growth. We show here that those periods are characterized by major changes in gene expression patterns. Thus, two samples taken from approximately the same growth phase may in fact not be comparable with respect to their transcription profiles. This problem may be exacerbated if one of the cultures is treated in such a way that the growth rate is, even slightly, affected, as that could perturb the synchronicity of sampling.

The use of marker genes enables the current chrono-transcriptomics experiment to be used as a background model for other more conventional single-time-point (stp) experiments. By identifying the chrono-transcriptomics sample most identical to an stp sample, one can determine the changes in gene expression likely to have been caused by growth effects and those that are due to, e.g., the condition applied. Furthermore, when examining an L. lactis strain that is severely retarded in growth, this analysis may be used to more accurately determine the approximate growth phase of the culture than what visual inspection of the growth curve would allow to.

**FIG 7:**
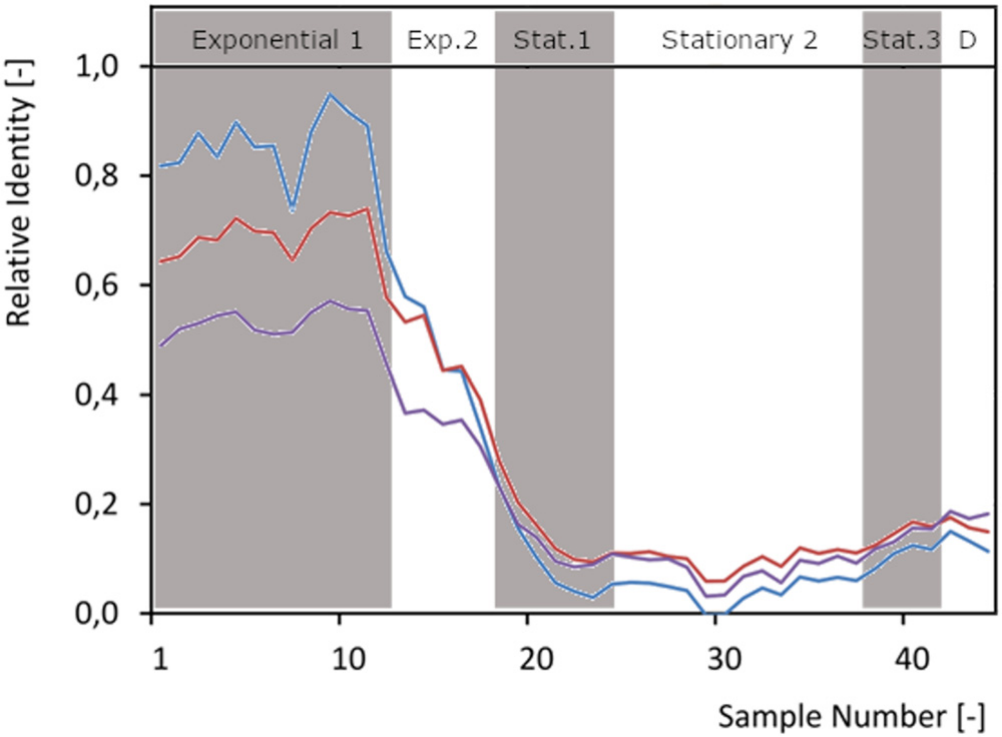
Use of the L. lactis chrono-transcriptome data set as a reference curve. Transcriptome data of L. lactis cells 0 min (blue) or 64 min (red) after induction of OpuA overproduction, and those of L. lactis cells overproducing the human protein presenilin 64 min after induction (violet) (17), were compared to the data at 45 time points of the chrono-transcriptome. Genes considered to be growth phase markers were used to correlate data between each of the stps and the entire growth curve. Gray and white bands indicate the different growth phases as indicated on the top axis. D, death.

Three DNA microarray data sets (17) were matched to samples from the chrono-transcriptomics experiment presented here (Fig. 7). In all these stp experiments, an L. lactis MG1363-derived strain was compared to the same strain that was induced to overproduce a membrane protein. The L. lactis cells overproducing the homologous protein OpuA, but sampled just prior to induction, greatly resembled cells around tp 10 in the L. lactis chrono-transcriptome. This is as expected since the cells were about to be induced around that point in growth and optical density. Culture growth became slightly affected approximately 64 min after induction; it lost a bit of the identity of an exponentially growing culture, and its overall gene expression profile slightly changed to that of a culture later in growth (Fig. 7). This observation was even more pronounced in a culture of cells overproducing the human protein presenilin, as indeed expected since these cells display a significant growth defect (17) (Fig. 7).

As most of the published transcriptional data sets are highly comparable to the data of the chrono-transcriptomics experiment described here, and since L. lactis cells are typically grown under roughly the same conditions (standing GM17 batch cultures at 30°C), gene expression information from the latter can be transposed to the experiments described in literature. This is especially relevant when only a small number of biological replicates are described in the latter. This high-density chrono-transcriptomics study can serve as a reference to clarify whether observed expression changes are due to (i) the treatment applied to cells, (ii) expression fluctuations that characterize the sampling period (as discussed below), or (iii) indirect effects such as those that derive from a growth rate change.

### Abrupt transient changes in gene expression.

**FIG 8:**
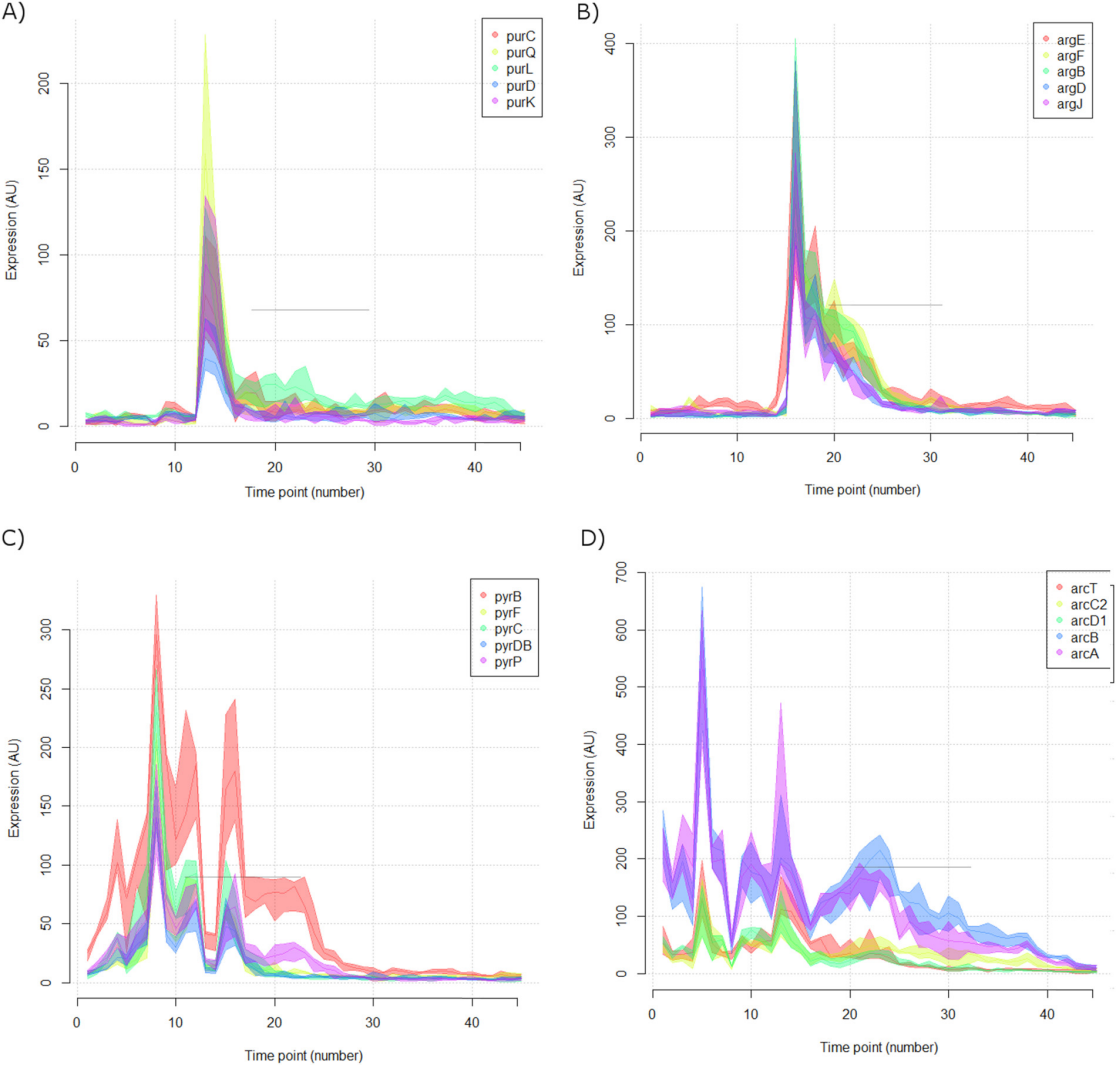
Certain L. lactis genes show abrupt and transient changes in gene expression. Expression profiles of genes representative of operons/regulons involved in purine (A), pyrimidine (C), and arginine (B and D) metabolism.

An important observation from this high-resolution chrono-transcriptome is that a fraction of the genes of L. lactis display abrupt and transient changes in gene expression, even during the middle of the seemingly monotonous exponential phase 1. Notably, even genes involved in central metabolism, such as those involved in purine and pyrimidine biosynthesis (18) and those involved in arginine metabolism (19), belong to this group (Fig. 8).

**FIG 9:**
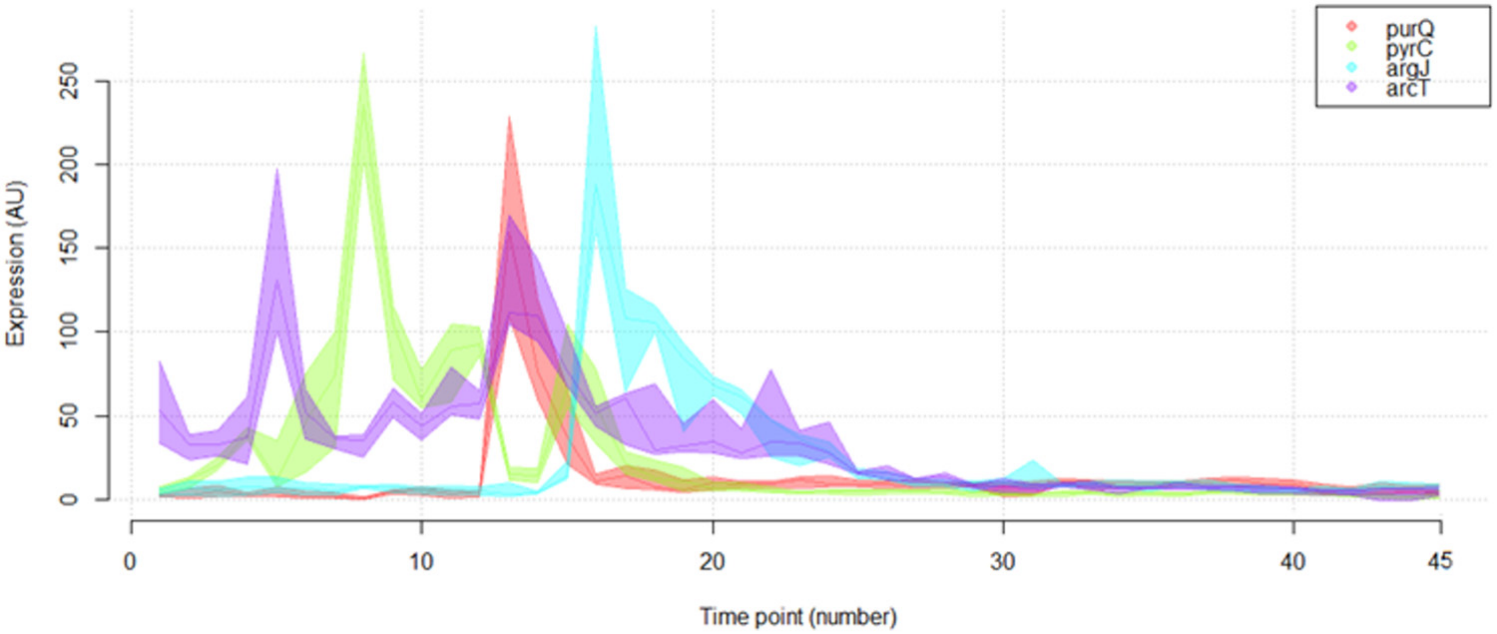
Expression of certain genes of L. lactis MG1363 is characterized by abrupt transient changes. The high-resolution chrono-transcriptome of an L. lactis MG1363 culture in GM17 at 30°C is shown. During growth, some genes sporadically assume abnormally high expression levels. This is particularly the case at a time point when standard sampling is generally performed, namely, at or around the mid-exponential phase of growth.

The behavior of these genes could possibly lead to misinterpretations in the analyses of stp transcriptome experiments in which, generally, a variant strain in which a gene is deleted or overexpressed is compared to its parental strain. Classically, these cultures are harvested around the mid-exponential growth phase, in which a minor difference in the growth of both strains could result in significant differences in the amounts of specific mRNAs. Thus, genes showing such transcription bursts could easily be falsely listed as being differentially expressed. Indeed, the reason that *arg*, *arc*, *pyr*, and *pur* genes are frequently observed as being affected in these types of experiments might be that these genes show such an irregular expression behavior especially during the exponential growth phase. By overlapping such expression patterns, one can conclude that there are only very short-lived intervals where the overall expression pattern remains constant (Fig. 9).

### Expression weighted properties.

**FIG 10:**
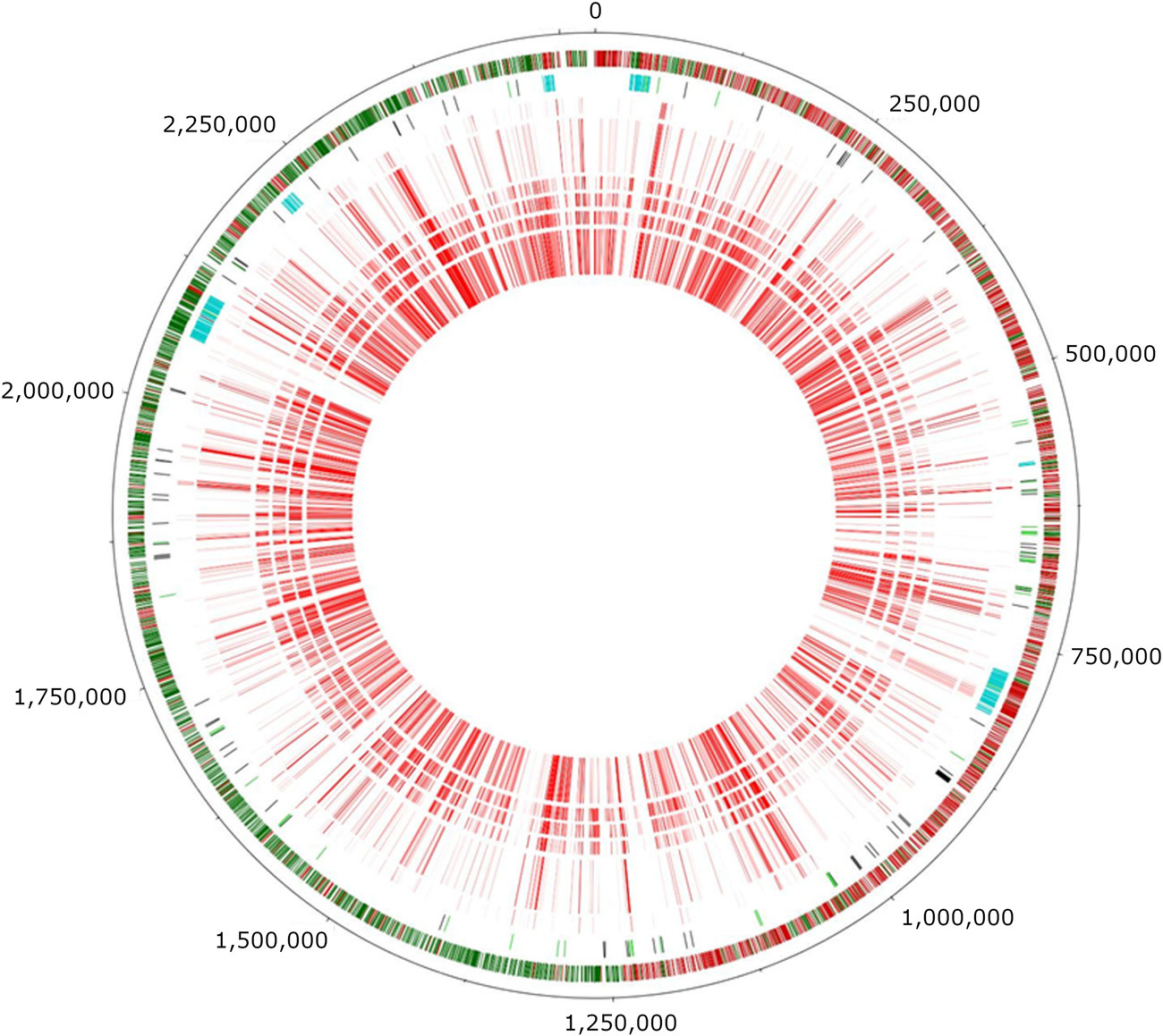
The chrono-transcriptome plotted onto a chart of the L. lactis MG1363 genome. The six innermost circles indicate the level of expression of genes in the different growth phases (exponential subphase 1 being the closest to the center; the intensity of the red color is proportional to the averaged level of transcription in each phase). The seventh circle depicts prophage remnants (blue), insertion sequence (IS) elements (green), and pseudogenes (black). The outermost circle depicts the orientation of genes (red when coded on the forward strand; green when present on the reverse strand).

By plotting gene expression levels on a map of the genome of L. lactis MG1363, conspicuously silent regions were highlighted; these corresponded to places where the genome has undergone recent rearrangements, notably areas rich in transposable elements or carrying bacteriophage genes (8) (Fig. 10). While most genes stop being transcribed during stationary subphase 3, some phage genes show an opposite behavior and are only transcribed when growth ceased. There is also a tendency throughout growth for genes close to the origin of replication to be expressed at higher levels than the average throughout the genome (Fig. 10). This could be a consequence of genes closer to the origin of replication having, on average, a higher copy number per cell, because they are replicated sooner than the others during the cell cycle.

**FIG 11:**
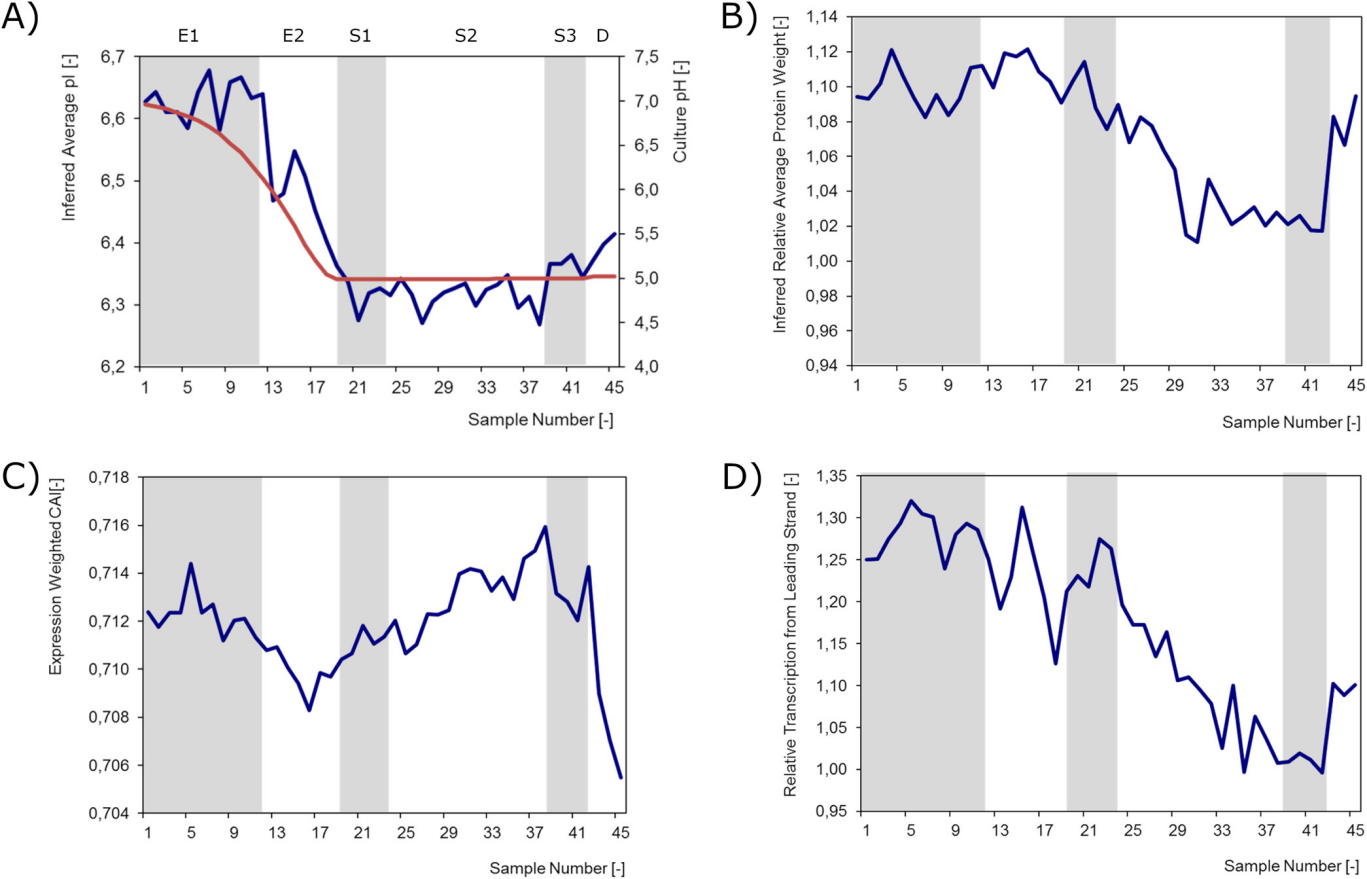
Expression weighted properties. (A) Average inferred pI of all proteins putatively produced at a given time point (blue) and culture pH (red). (B) Average inferred molecular weight of all proteins putatively produced at a given moment relative to the genomic averaged inferred value (32.6 kDa). (C) Average inferred weighted codon adaptation index (CAI; genomic average is 0.695). (D) Relative transcription from the leading strand, normalized for the overrepresentation of genes coded on that strand. For all panels, gray and white bands indicate, from left to right and in accordance with the growth phases described in the text, exponential phase 1, exponential phase 2, stationary phase 1, stationary phase 2, stationary phase 3, and death phase.

It was also observed that throughout the chrono-transcriptome a bias exists toward the expression of genes encoding proteins with predicted pIs lower than that of the average value of all proteins encoded in the genome of L. lactis MG1363 (Fig. 11A). This predisposition is further accentuated during growth and correlates with the decrease of the pH of the medium. Possibly, L. lactis has evolved mechanisms to cope with lactate, its main metabolic by-product, by producing comparatively low-pI proteins when environmental pH decreases. This seems to constitute a possible crucial adaptation, since it has been observed that there is a high correlation between external and internal pH for L. lactis (20, 21).

By extrapolating the data to translation, assuming that protein synthesis is roughly proportional to mRNA levels, one can infer that the average size of proteins predicted to be synthesized at any given moment is higher than the average size of all proteins coded in the genome. During stationary subphase 3, cells seem to greatly diminish the average size of synthesized proteins (Fig. 11B). This might represent a mechanism accounting for nutrient scarcity and a way to optimize resources.

A tendency was also observed for L. lactis to express genes with a better-than-the-average codon adaptation index (CAI) throughout growth (Fig. 11C). This trend, at the level of transcription, is quite constant during growth, being only slightly alleviated in the death phase, presumably because translation levels are so low that codon usage is not likely to be a constraint anymore.

The L. lactis chrono-transcriptomics results reveal a striking correlation in time between gene strand bias and transcription intensity. Genes coded on the leading strand (the transcription sense strand is the same as the replication leading strand) are, on average, more expressed than those encoded on the lagging strand (transcription sense strand is the same as the replication lagging strand). One could hypothesize that this represents an adaptation to deal with the interference between DNA replication and RNA transcription, in particular with respect to the direction of one process relative to the other (22, 23). Indeed, the observed bias is more pronounced in fast-growing cells, in the exponential phase, but minimal during stationary phase and onwards when both replication and transcription levels diminish (Fig. 11D). It has been disputed whether essentiality or expressiveness could drive gene strand bias (see, for example, reference 24), although very likely both are relevant.

### Perspectives.

The transcriptomics data obtained in this work represent a repository that can be mined by the LAB research community, similarly to that comprised by LAB genome data. We performed a global analysis on the whole data set and a more in-depth survey into major fields of interest of L. lactis research. All raw data and the normalized gene expression profiles are shared together with this text, as supplemental material, and should allow rapid progress in both fundamental and application-oriented research on L. lactis.

## MATERIALS AND METHODS

### Bacterial strain and growth conditions.

Lactococcus lactis subsp. *cremoris* MG1363 (25), from the same −80°C aliquot as the one that was used for the sequencing of the genome of this strain (8), was grown in M17 medium (Difco Laboratories, Detroit, MI) supplemented with 0.5% (wt/vol) glucose (Acros Organics, Geel, Belgium) at 30°C. A chemostat of 16 L (working volume of 12 L) was used, and homogeneity of the culture was maintained through mild stirring (30 rpm). The medium pH was monitored with a built-in probe, and growth was followed by measuring the optical density at 600 nm (OD_600_). Prewarmed medium was inoculated with a 1/100 volume of an L. lactis MG1363 GM17 culture maintained in the exponential phase of growth by means of consecutive dilutions, during a period of at least 48 h. Reproducibility of the procedure was verified by repeating the above-described fermentation procedure 2 times. Cultures and inocula were grown in media from the same batch. Samples from all inocula and from the cultures at the end of the experiment were plated, examined under a microscope, and regrown in microtiter plates to check for potential contamination and plated to check cell viability by counting CFU (data not shown). Glucose concentration was measured via an enzymatic assay using a d-glucose kit (R-Biopharm, Darmstadt, Germany) according to the manufacturer’s instructions. Measurements were done in duplicate on cell-free supernatant samples obtained during fermentation.

### Sampling and RNA isolation.

The equivalent of 10 OD units of volume (volume [in milliliters] × OD_600_) was taken in duplicate every 15 min during 12 h. Additionally, triplicate samples (15 min apart) were taken at each time point corresponding to 24, 36, and 48 h after inoculation. Cells were pelleted in 50-mL Greiner tubes in an Eppendorf 5810R centrifuge (Eppendorf AG, Hamburg, Germany; 1 min, 10,000 rpm, and 4°C) and resuspended in 0.5 mL of T_10_E_1_ (10 mM Tris-HCl, 1 mM Na_2_-EDTA [pH 8.0]), prepared with diethylpyrocarbonate (DEPC)-treated deionized water before transfer to 2-mL screw-cap tubes. The resuspended cells were immediately frozen in liquid nitrogen and then kept at −80°C.

For RNA isolation, 0.5 g of glass beads (∼100 μm in diameter), 50 μL of 10% sodium dodecyl sulfate (SDS), 500 μL of premixed phenol-chloroform-isoamyl alcohol (25:24:1), and 175 μL of Macaloid (Bentone MA; Elementis Specialties, Inc., Hightstown, NJ) suspension (prepared as described in reference 26) were added to the thawed cells in the screw-cap tube. Cells were disrupted using 2 cycles of 45 s of bead beating with a 1-min interval on ice. The cell lysate was cleared by centrifugation in an Eppendorf 5417R centrifuge (Eppendorf AG) (10 min, 10,000 rpm, and 4°C), after which the upper phase was extracted with 500 μL of chloroform-isoamyl alcohol (24:1). The two phases were resolved by centrifugation (10 min, 10,000 rpm, and 4°C), and total RNA was isolated from the aqueous phase using a High Pure RNA isolation kit (Roche Molecular Biochemicals, Mannheim, Germany), according to the manufacturer’s instructions. The RNA concentration was determined with a NanoDrop ND-1000 spectrophotometer (NanoDrop Technologies, Wilmington, DE); RNA quality was assessed using an Agilent Bioanalyzer 2100 with RNA 6000 LabChip (Agilent Technologies Netherlands BV, Amstelveen, the Netherlands).

### DNA microarray procedure.

**FIG 12:**
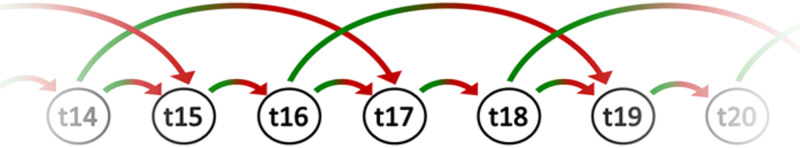
Hybridization scheme. Synthesized cDNA obtained from the purified total RNA from each sample, labeled with either Cy3 or Cy5 (according to the green or the red hedge of the arrows, respectively), was hybridized according to the indicated dye-swap strategy on 3 DNA microarray slides.

Synthesis of complementary DNA (cDNA) was performed using a Superscript III reverse transcriptase kit (Invitrogen, Carlsbad, CA). Incorporation of amino allyl-modified dUTPs during cDNA synthesis allowed Cy3/Cy5 labeling with a CyScribe postlabeling kit (Amersham Biosciences, Piscataway, NJ) according to the supplier’s instructions. All intermediate and final purifications of either labeled or unlabeled cDNA were performed with a NucleoSpin Extract II kit (Clontech Laboratories, Mountain View, CA) according to manufacturer’s instructions except while purifying unlabeled cDNA, where 80% ethanol was used in a second washing step and 0.1 M sodium bicarbonate (pH 9.0) was used as the elution buffer. The hybridization of all samples followed the scheme depicted in Fig. 12. Thus, the transcript levels for every gene were measured at least 6 times for every time point (3 slides per time point and at least duplicate spots for each gene).

Hybridization to the probes spotted onto the L. lactis MG1363 mixed amplicon and oligonucleotide DNA microarray slides, covering 2,308 of the 2,435 predicted open reading frames (ORFs), was done using the Slidehyb no. 1 hybridization buffer (Ambion Biosystems, Foster City, CA) for 16 h at 45°C. After hybridization, slides were washed for 5 min in 2× SSC (150 mM NaCl and 15 mM trisodium citrate) with 0.5% SDS, 2 times for 5 min in 1× SSC with 0.25% SDS and 5 min in 1× SSC with 0.1% SDS. All washing steps were performed at 30°C with preheated buffers. The washing buffers were removed via centrifugation (Eppendorf 5810R, 2 min, and 2,000 rpm). The DNA microarray slides were scanned using a GenePix Autoloader 4200AL confocal laser scanner (Molecular Devices Corporation, Sunnyvale, CA).

The resulting images were analyzed using the ArrayPro Analyzer 4.5 software (Media Cybernetics, Silver Spring, MD). Signal intensities were quantified for each spot on the DNA microarray slides after subtracting the background intensities, which were determined for each spot by reading the signals in the regions that separated diagonal spots. Signals were initially normalized and scaled via locally weighted scatterplot smoothing (LOWESS) using MicroPrep software (27), after which a dimensioning-noise-amplitude (D-N-A) scaling was performed, as described above.

### Output visualization.

Gene expression profiles were studied and depicted using R 2.15.1 software packages (The R Project for Statistical Computing [http://www.r-project.org/]). Gene expression plots illustrate the range of expression signals obtained for each gene at each time point, of all the duplicate spots from the 3 microarray slides where each time point sample preparation was loaded; the darker line within each range represents the median value. PCA data were equally obtained using R 2.15.1 software packages. The genome circular plot in Fig. 10 was obtained using DNAPlotter (28).

### Matching microarray experiments against the chrono-transcriptome reference curve.

Microarray signals from three DNA data sets (17) were modified according to the D-N-A procedure described above. Signals from the 45 genes considered to be markers of growth phase (see above) were chosen and plotted against those from each time point from the chrono-transcriptome, and the correlations were used as a measure of the relative identity of gene expression between samples. For clarity purposes, results were normalized so that 1 would be the correlation of any sample against itself and 0 the correlation of the two most different samples in this analysis.

### Expression weighted properties.

The whole-genome expression data obtained through this study were used to infer macroadaptation trends throughout the growth curve. Thus, we were interested in the genome position and direction of genes being mostly transcribed, their codon optimization, and whether there was any general trend regarding the proteins being (putatively) translated. Since nutrient availability/starvation and pH played critical roles in this experiment, we set out to infer whether the first might modulate the molecular weight and the latter the pI of the proteins being produced at each time point. Clearly, the assumption is that transcript levels correlate well with the level of translation of the respective protein at that moment.

The general position of the genes being most transcribed was examined using the genome plotting, as described above. The other whole-cell properties (average protein molecular weight (MW) and pI, average codon adaptation index, and relative sense transcription) were calculated for each time point by weighting the associated value of each gene with its expression level. The weighting formulas for each time point are as follows.

For inferred average pI (equation 1), a value of 6.5 means that at that point in time, cells produce proteins with an average pI of 6.5.

(1) $$\mathrm{Inferred average pI}=\frac{\sum_{i=0}^{n}\mathrm{expression leve}l_{i}\times pI_{i}}{\sum_{i=0}^{n}\mathrm{expression leve}l_{i}}$$

For inferred relative average protein weight (equation 2), *n* is number of genes represented in the microarrays. A value of 1.10 means that at that time point, cells produce proteins with an MW that is, on average, 10% higher than the average of all proteins encoded in the genome of L. lactis MG1363.

(2) $$\mathrm{Inferred relative average protein weight}=\frac{\frac{\sum_{i=0}^{n}\mathrm{expression leve}l_{i}\times MW_{i}}{\sum_{i=0}^{n}\mathrm{expression leve}l_{i}}}{\frac{\sum_{i=0}^{n}MW_{i}}{n}}$$

In expression weighted CAI (equation 3), a gene CAI varies between 0 and 1, depending on whether the gene uses rare codons or frequent codons, respectively (29). On average, genes in the L. lactis MG1363 genome have a CAI of 0.695. An expression weighted CAI of, e.g., 0.715 means that the organism is transcribing genes with a better than average codon usage.

(3) $$\mathrm{Expression weighted CAI}=\frac{\sum_{i=0}^{n}\mathrm{expression leve}l_{i}\times \mathrm{CA}I_{i}}{\sum_{i=0}^{n}\mathrm{expression leve}l_{i}}$$

For relative transcription from the leading strand (equation 4), *i* represents genes for which the transcription sense strand is the same as the replication leading strand and *n* the total number of those genes, and *j* represents genes for which the transcription sense strand is the same as the replication lagging strand and *m* the total number of those genes. Only genes represented in the DNA microarrays were considered. A value of, e.g., 1.20 means that genes coded on the leading strand at a certain time point are, on average, 20% more expressed that the other genes, even after normalizing for their genomic overrepresentation (1863 versus 439).

(4) $$\mathrm{Relative transcription from leading strand}=\frac{\frac{\sum_{i=0}^{n}\mathrm{expression leve}l_{i}}{n}}{\frac{\sum_{j=0}^{m}\mathrm{expression leve}l_{j}}{m}}$$
