# Supplementary material for: High-Resolution Chrono-Transcriptome of Lactococcus lactis Reveals That It Expresses Proteins with Adapted Size and pI upon Acidification and Nutrient Starvation
Source: Appl Environ Microbiol. 2022 Apr 13;88(9):e02476-21. doi: 10.1128/aem.02476-21 (PMC9088255; doi:10.1128/aem.02476-21)
Supplement: Supplemental file 1 — Description of Data Set S1. Download aem.02476-21-s0001.pdf, PDF file, 0.06 MB [file aem.02476-21-s0001.pdf]

Legend to Supplemental Tables 1 and 2 (as Excel file)

Table 1: Signal Raw Data

Table 2: D-N-A Scaled Data

Sheet 3: Signal-TP Mapping (Factors*)

)* These Factors can be used in T-rex, a comprehensive tool for the analysis of gene expression data (http://genome2d.molgenrug.nl)
